# Association between Soft Drink Consumption and Aggressive Behaviour among a Quarter Million Adolescents from 64 Countries Based on the Global School-Based Student Health Survey (GSHS)

**DOI:** 10.3390/nu12030694

**Published:** 2020-03-05

**Authors:** Zumin Shi, Ahmed Malki, Abdel-Salam G Abdel-Salam, Jianghong Liu, Hatem Zayed

**Affiliations:** 1 Human Nutrition Department, College of Health Sciences, QU Health, Qatar University, 2713 Doha, Qatar; 2 Department of Biomedical Sciences, College of Health Sciences, QU Health, Qatar University, 2713 Doha, Qatar; ahmed.malki@qu.edu.qa (A.M.); hatem.zayed@qu.edu.qa (H.Z.); 3 Department of Mathematics, Statistics and Physics, College of Health Sciences, QU Health, Qatar University, 2713 Doha, Qatar; abdo@qu.edu.qa; 4 University of Pennsylvania School of Nursing, Philadelphia, PA 19104, USA; jhliu@nursing.upenn.edu

**Keywords:** soft drink, aggression, adolescents

## Abstract

Soft drink consumption has become a significant public health concern that is associated with various adverse health outcomes. We aim to examine the association between soft drink consumption and aggressive behavior among adolescents. We used open access data from 79 studies in 64 countries, including 263,890 adolescents aged 12–18 years who completed the global school-based student health survey (GSHS). Self-reported data on past 30-day carbonated soft drink consumption (number of times per day) and past 12-month physical fighting were utilized for analysis. Of the 263,890 participants (48% boys) aged 12–18 years, the weighted mean frequency of soft drink consumption varied from 0.5 in Kiribati to 2.5 times/day in Surname, while the weighted prevalence of frequent aggressive behavior varied from to 2.7% in Laos to 49.2% in Tuvalu. We found that each increment of soft drink consumption (time/day) was associated with an 11% (95%CI 10–13%) increase of the likelihood of frequent physical fighting. This result remained significant after adjusting for various covariates. In this large pooled sample of multinational data, there is a significant positive association between soft drink consumption and aggressive behavior among adolescents. Reducing soft drink consumption may help reduce aggressive behavior, a major risk factor for violence.

## 1. Introduction

Aggressive behavior is a major concern among adolescents and causes injury [1]. The prevalence of aggressive behavior varies between countries substantially [2,3]. Data from a study conducted in 79 countries suggest that the prevalence of frequent physical fighting (≥4 times during the past 12 months) was 10.4% in boys and 2.7% in girls [3].

Diet plays an important role in maintaining psychological wellbeing [4]. A Western dietary pattern is associated with increased mental health and behavioral problems. Soft drink consumption has been shown to be associated with mental health problems [5,6]. Some of the previous studies focused on the association between soft drink consumption and depression, suicidal ideation among adolescents [5,6,7]. Although the mechanisms are not fully understood, a positive association between soft drink consumption and these mental health problems has been reported in both developing and developed countries [5,6,7]. Most of the studies hypothesized that inflammation or oxidative stress caused by soft drink mediates the link between soft drink consumption and mental health problems.

Past studies on soft drink consumption and behavior have largely focused on internalizing behavior. A limited number of studies have examined the relationship between soft drink consumption and aggressive behavior among adolescents [8,9,10]. In the Youth Risk Behavior Survey in the USA, Solnick et al. found that soft drink consumption was positively associated with aggressive behavior [9]. In 26 industrialized countries, sugar consumption is positively associated with risk behavior, including physical fighting, mainly due to a sugary drink rather than sweets [10]. It is unknown whether this association exists in other countries with different culture and eating behaviors. Understanding the role of soft drink in the burden of injury and violence has its public health significance, as diet is a modifiable factor.

The aim of the study was to assess the association between soft drink consumption and aggression among school adolescents aged 12–15 years in 64 countries/regions participated in the global school-based student health survey (GSHS) between 2007 and 2016.

## 2. Materials and Methods

### 2.1. Study Design and Sample

The global school-based student health survey (GSHS) is a collaborative surveillance project between WHO, USA CDC, and respective countries. It was designed to measure and assess the behavioral risk factors and protective factors among school adolescents. The details of the survey can be found on the GSHS website (https://www.who.int/ncds/surveillance/gshs/en/). In short, the survey used a two-stage probability sampling method to recruit participants. In the first stage, schools were selected. In the selected schools, classrooms with students aged 12–18 were randomly selected at the second stage. All the students in the selected school classrooms were all invited to participate in the study regardless of age. In each country, ethical approval of GSHS was obtained from both a national government (the Ministry of Health or Education) and an institutional review board or ethics committee. Informed consent was obtained from the students, parents, and/or schools. Data were weighted for non-response and the survey design.

In the current study, publically available data from 64 countries were used. We retrieved the data from the WHO website. The surveys were conducted between 2007 and 2016. For each country, we selected nationally representative data. If the nationally representative data were not available, regional data were used.

### 2.2. Outcome Variable: Aggression

Aggressive behavior was assessed by the question “During the past 12 months, how many times were you in a physical fight?”. Frequent fighting was defined as physical fight ≥3 times during the past 12 months [2]. In the study, we used the involvement of frequent physical fighting as an indicator of aggressive behavior.

### 2.3. Exposure Variable: Soft Drink Consumption

Soft drinks consumption was assessed by the question “During the past 30 days, how many times per day did you usually drink carbonated soft drinks? (1) I did not drink (2) less than 1 time per day; (3) 1 time per day; (4) 2 times per day; (5) 3 times per day; (6) 4 times per day; (7) 5 or more times per day”. In the analysis, soft drink consumption was recoded to a continuous variable (times/day). For those reported drinking soft drink <1 time per day, 5 or more times per day, we assigned a value of 0.5 and 5.5 times/day, respectively. We also categorized the consumption into five levels: none, <1 time/day, 1 time/day, 2 times/day, and ≥3 times/day.

### 2.4. Covariates

We used food insecurity as an indicator for socioeconomic status. It was assessed by the question “During the past 30 days, how often did you go hungry because there was not enough food in your home? (1) Never; (2) rarely/sometimes; (3) most of the time/always”. Physical activity was categorized as sufficient or in sufficient based on whether the participants engaged in >5 days of at least 60 min physical activity in a week. Smoking (yes/no) was defined as the use of any form of tobacco on at least one day in the past 30 days. The frequency of fruit and vegetable intake during the past 30 days was assessed. Fast food consumption was assessed by the question “During the past 7 days, on how many days did you eat food from a fast food restaurant?”. In the analysis, the consumptions were recoded into times/day (fruit and vegetable) or days/week (fast food). Students were asked about the number of days with physical activity of at least 60 min during the past 7 days. Sufficient physical activity was defined as engaged in ≥5 days of at least 60 min of physical activity in a week. Body weight and height were self-reported. BMI was calculated and used to define nutritional status (underweight, normal, overweight, and obese) using the International Obesity Task Force (IOTF) criteria [11,12].

### 2.5. Data Analyses

The descriptive statistics (e.g., mean and proportion) were calculated using svy mean or svy proportion in Stata to take into account the complex study design. The association between soft drink consumption and aggressive behavior was assessed using multivariable logistic regression in each survey with svy command in Stata. Soft drink consumption (times/day) was treated as a continuous variable in the multivariable logistic regression model. The model adjusted for age, gender, smoking, sufficient physical activity, food insecurity, and fast-food, fruit, and vegetable consumption. In all the analyses, for each covariate with a missing value we assigned a specific group to the variable other than excluding the participants or conducted multiple missing imputations.

To combine the estimates each country, the random effect meta-analysis method was used. Results were visually presented in a forest plot. Heterogeneity between groups was tested by Cochran’s Q tests. Subgroup meta-analysis was conducted by region, study level soft drink consumption and prevalence of aggressive behavior (by tertiles).

Furthermore, unweighted individual data were used to assess the association between soft drink consumption and aggressive behavior. In these analyses, soft drink consumption was treated as a categorical variable. A set of multivariable logistic regression models were used: Model 1 adjusted for age and gender; Model 2 further adjusted for smoking, alcohol drinking, sufficient physical activity (yes or no), intake of fruit and vegetable (times/day); Model 3 further adjusted for BMI status. To test the interaction between soft drink consumption with gender, physical activity, BMI status, and smoking, we put the product term of them in the multivariable logistic model and excluded those with missing values.

All the analyses were conducted using Stata 16 (Stata Corporation, College Station, TX, USA). Statistical significance was considered when *p*-value < 0.05 (two sided level-of-significance).

## 3. Results

### 3.1. Sample Characteristics

In 79 studies of 263,890 adolescents (127,060 boys and 136,830 girls) in 64 countries/regions, the weighted prevalence of aggressive behavior varied from 2.7% in Laos to 49.2% in Tuvalu (Figure S1). Similarly, the weighted mean frequency of soft drink consumption also varied by countries (from 0.5 in Kiribati to 2.5 times/d in Suriname).

Table 1 shows the sample characteristics across different soft drink consumption levels. The prevalence of smoking, alcohol drinking increased with the increase of soft drink consumption. Among those consumed soft drink ≥3 times/d, the prevalence of smoking and alcohol drinking was 18.7% and 30.3%, respectively. Across the soft drink consumption levels from low to high, the consumption of fruit, vegetable, and fast food increased. The crude prevalence of aggressive behavior across soft drink consumption of none, <1 time/day, 1 time/day, 2 times/day, and ≥3 times/day was 14.1%, 16.2%, 17.2%, 20.5%, and 26.4%, respectively.

### 3.2. Association Between Soft Drink Consumpiton and Aggressive Behaviour

In the multivariable logistic regression model, smoking, alcohol drink and food insecurity were positively associated but age was inversely associated with aggressive behavior. Girls were less likely to report aggressive behavior than boys (Figure S2). 

**Figure 1:**
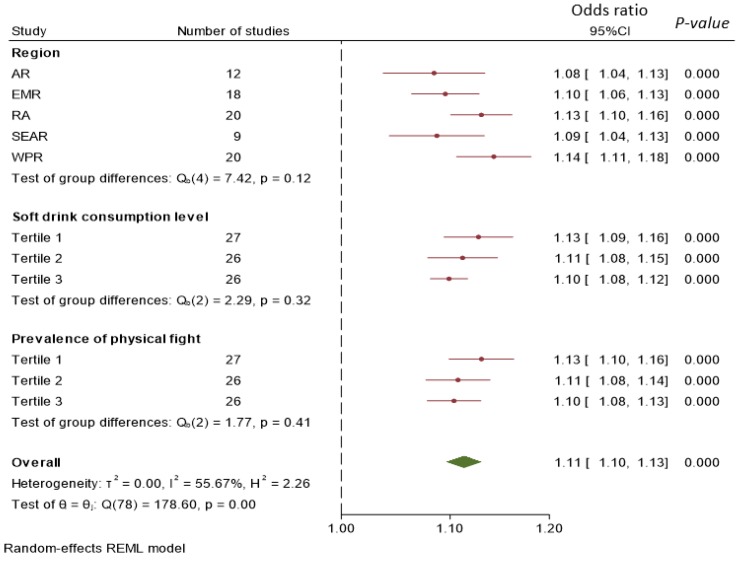
Forest plot of subgroup meta-analysis of the association between soft drink consumption and aggressive behavior. Abbreviation for regions: AR (African region), EMR (Eastern Mediterranean), RA (Region of Americas), SEAR (South East Asia Region), and WPR (Western Pacific Region). Values are odds ratio (OR) and 95% confidence interval (CI).

After adjusting for potential confounders, for each time/day increase of soft drink consumption, there was an 11% (10–13%) increase of the self-reported aggressive behavior in the aggregated data from all the countries (Figure 1). There was no significant difference by regions, study level soft drink consumption and prevalence of aggressive behavior. In country specific analysis, the highest odds ratio (OR) was found in Niue (Figures S3–S7). There was a strong heterogeneity between countries in each region. In the region of the Americas, most of the studies suggest a positive association between soft drink consumption and aggressive behavior.

Using unweighted individual data, there was a significant dose response relationship between soft drink consumption and aggressive behavior (Table 2). After adjusting for age and gender, compared with non-consumers of soft drink, those consumed soft drink ≥3 times/day had 114% (95%CI 104–121%) increased likelihood of reporting frequent aggressive behavior. In the fully adjusted model, using none consumers as the reference group, across soft drink consumption levels of none, <1 time/d, 1 time/d, 2 times/d, and ≥3 times/d the OR (95%CI) for aggressive behavior were 1.08 (1.05–1.12), 1.09 (1.06–1.13), 1.22 (1.17–1.26), and 1.46 (1.41–1.52), respectively.

There was a significant interaction between soft drink consumption and smoking and physical activity. The association between soft drink consumption and aggressive behavior was stronger among non-smokers and those with sufficient physical activity than smokers and those with insufficient physical activity (Table 3). No significant interactions between soft drink consumption, gender and BMI status were found.

Among those with healthy lifestyle (non-smoking, had sufficient physical activity, and non-alcohol drinking), soft drink consumption was associated with aggressive behavior; across soft drink consumption levels of <1 time/d, 1 time/d, 2 times/d, ≥3 times/d, the OR for aggressive behavior were 1.16 (1.06–1.28), 1.30 (1.17–1.45), 1.16 (1.02–1.31), and 1.42 (1.26–1.60), respectively.

## 4. Discussion

In this cross-sectional study of a quarter million adolescents in 64 countries, soft drink consumption was positively associated with aggressive behavior in both genders. The association between soft drink consumption and aggressive behavior was stronger among non-smokers and those with sufficient physical activity. There are regional differences on the association between soft drink consumption and aggressive behavior. Substantial difference of soft drink consumption was observed in the study.

### 4.1. Comparison with Previous Studies

Although comparable results to our findings related to a positive association between soft drink consumption and aggressive behavior were reported in the USA among youth [9], Slovakia [8], and 26 industrialized countries [10], we for the first time categorized more studies from low and middle-income countries. This allows us to limit some of the confounding effects common in high-income countries (e.g., stress and high-energy dense food). There seems to be no significant difference in the association between soft drink consumption and aggressive behavior by country level income. However, there was significant heterogeneity among countries. The OR for aggressive behavior associated with soft drink consumption varied from 0.86 (95% CI 0.74–1.01) in Mozambique to 1.43 (95%CI 1.08–2.89) in Niue. The heterogeneity could not be explained by the country level soft drink consumption and prevalence of aggressive behavior (Figure 1). It is unknown whether different types of soft drink consumed in different countries may partly explain the difference. The use of types of sugar (e.g., high-fructose corn syrup (HFCS) vs. sucrose) to produce soft drink may contribute to the difference. Food culture difference may also explain the heterogeneity. Genetic determinants play critical roles in modulating aggressive behavior [13,14,15]. Thus, the heterogeneity observed in our study could be partly due to the difference of genetic background in different countries. Existing evidence suggests there are interactions between genetics and sugar-sweetened beverage consumption on health outcomes (e.g., obesity) [16].

The weak positive association between fruit and vegetable intake with aggressive behavior is consistent with a study from the USA [9]. Further studies are needed to elucidate the association between overall dietary patterns and aggressive behavior. Unhealthy lifestyles often cluster and affect aggressive behavior [17]. It is worth noting that among those with a healthy lifestyle (e.g., non-smoking, no alcohol drinking, and sufficient physical activity) in our study, there was still a strong positive association between soft drink consumption and aggressive behavior. It is less likely that the link was confounded by these lifestyle factors. Similar to other studies [18,19], we found that obesity, smoking, and alcohol drinking was positively associated with aggressive behavior.

### 4.2. Potential Mechanisms

Although the exact mechanisms linking soft drink consumption and aggressive behavior remain to be studied, several hypotheses can be made. Firstly, high consumption of sugar can increase the level of oxidative stress and inflammation [20]. These changes have been shown to be associated with mental health problems [21]. In a study of Wistar rats, it has been shown that carbonated soft drinks can induce oxidative stress and alter the expression of certain genes that are associated with brain activity [22]. Secondly, soft drink is an important component of the western diet. It has been shown that the Western diet is related to mental health problems and externalizing behavior [4]. Gut microbiota is a mediator for the link between modern diet and many chronic diseases including mental health problems [23,24,25]. Several factors may mediate the association between soft drink consumption and aggressive behavior. Findings from the Health Behavior in School-Aged Children (HBSC) study suggest that daily nervousness and irritability mediated the association between soft drink consumption and aggressive behavior among adolescents in Slovakia [8]. Some soft drink contains caffeine and caffeine has been linked to insufficient sleep, nervousness, and impulsivity and risk taking in children and adolescents [26]. In the International Study of Childhood Obesity, Lifestyle, and the Environment (ISCOLE) [27] and other studies [28,29], soft drink consumption has been linked to shorter sleep duration. Existing evidence suggests that poor sleep is a risk factor for aggressive behavior [30,31]. Thus, the link between soft drink consumption could be mediated by short sleep duration.

High consumption of HFCS in beverages may play a role in the epidemic of obesity [32]. One of the proposed mechanisms is based on the fact that fructose is metabolized differently than glucose in ways that favor de novo lipogenesis, which increase total body fat and do not stimulate insulin secretion or enhance the production of leptin, both afferent signals in the regulation of food intake and body weight [32]. In our data, soft drink consumption was positively associated with obesity. Although the previous study suggested that body size was associated with aggressive behavior, overweight/obesity is unlikely a strong confounding factor between soft drink consumption and aggressive behavior in the present study as the association between soft drink consumption and aggressive behavior was observed in all BMI categories. Furthermore, with the adjustment for BMI the effect estimates did not change.

Soft drink consumption clusters with other unhealthy lifestyles including smoking, alcohol drink, and sedentary activity [33]. In our study, the association between soft drink consumption and aggressive behavior was stronger among non-smokers and those with sufficient physical activity than their counterparts (Table 3). It could be that the association between soft drink consumption and aggressive behavior was less confounded by other lifestyle factors among those with a relatively healthy lifestyle. Furthermore, it could also suggest that the adverse effects of soft drink consumption cannot simply be mitigated by other healthy lifestyles (e.g., fruit and vegetable consumption).

### 4.3. Strength and Limitation

The main strength of our study is the large number of participants (*n* = 263,890) and the comprehensive methods of collecting data through a well-designed survey aiming to pinpoint on the possible correlation between lifestyle factors and health conditions. In addition, the study represents global dimension of behaviors, including 64 different countries with different food cultures, life style, and habits, encompassing five WHO regions (i.e., African region, Eastern Mediterranean, Region of Americas, South East Asia Region, and Western Pacific Region). The consistent findings of the relationship between soft drink consumption and aggressive behavior in most of the countries is less likely due to chance or biased by unmeasured confounding factors. The main limitation of the study is its cross-sectional study design and a lack of quantitative measures of soft drink consumption other than the frequency of intake. Furthermore, unmeasured residual confounding variables, such as socioeconomic status and parenting style, could potentially contribute to the relationship between soft drink consumption and aggressive behavior.

### 4.4. Public Health Implications

Childhood aggressive behavior is a major predisposition to adolescent delinquency and later adult violence. Thus, understanding its risk factor may help diminish adult violence. Overall, population attributable risk of aggressive behavior due to soft drink consumption may vary substantially by countries. In countries with high soft drink consumption, the benefit of reducing soft drink consumption might be substantial based on the study. Our findings may shed light on the practical implications from a public health perspective in increasing awareness on the harmful consequences of soft drink consumption on behavioral and mental health outcomes, such as aggressive behavior. This initial finding will serve as the first step in providing future interventions.

In conclusion, soft drink consumption was positively associated with aggressive behavior independent of sociodemographic and lifestyle factors. Strategies are needed to reduce the consumption of soft drink to prevent intentional injuries related to the aggressive behavior among adolescents. Further studies may provide further insight in the molecular mechanisms linking soft drink consumption and aggressive behavior.

## Figures and Tables

**Table 1:** Sample characteristics by levels soft drink consumption (*n* = 263,890): the global school-based student health survey (GSHS) ^1^.

	None	<1 Time/Day	1 Time/Day	2 Times/Day	≥3 Times/Day	*p*-Value
*n*	49,917	84,391	56,244	34,012	39,326	
Age (years), mean (SD)	14.7 (1.5)	14.7 (1.5)	14.6 (1.5)	14.5 (1.4)	14.5 (1.4)	<0.001
Sex						<0.001
Male	23,251 (46.6%)	40,800 (48.3%)	27,441 (48.8%)	16,426 (48.3%)	19,142 (48.7%)	
Female	26,666 (53.4%)	43,591 (51.7%)	28,803 (51.2%)	17,586 (51.7%)	20,184 (51.3%)	
Food insecurity	3744 (7.6%)	4760 (5.7%)	3670 (6.6%)	2093 (6.2%)	3279 (8.4%)	<0.001
Aggressive behavior	7090 (14.2%)	13,643 (16.2%)	9930 (17.7%)	6977 (20.5%)	10,373 (26.4%)	<0.001
Smoking						<0.001
No	42,381 (84.9%)	69,136 (81.9%)	43,750 (77.8%)	25,916 (76.2%)	27,873 (70.9%)	
Yes	3659 (7.3%)	8670 (10.3%)	6268 (11.1%)	4499 (13.2%)	7343 (18.7%)	
Not assessed	3150 (6.3%)	5417 (6.4%)	5334 (9.5%)	2998 (8.8%)	3199 (8.1%)	
Missing	727 (1.5%)	1168 (1.4%)	892 (1.6%)	599 (1.8%)	911 (2.3%)	
Alcohol drinking						<0.001
No	32,310 (64.7%)	51,629 (61.2%)	31,486 (56.0%)	18,315 (53.8%)	17,767 (45.2%)	
Yes	4951 (9.9%)	13,160 (15.6%)	9602 (17.1%)	7444 (21.9%)	11,916 (30.3%)	
Not assessed	11,517 (23.1%)	17,537 (20.8%)	13,429 (23.9%)	7116 (20.9%)	8004 (20.4%)	
Missing	1139 (2.3%)	2065 (2.4%)	1727 (3.1%)	1137 (3.3%)	1639 (4.2%)	
Sufficient physical activity						<0.001
No	35,339 (70.8%)	60,132 (71.3%)	40,554 (72.1%)	24,174 (71.1%)	26,491 (67.4%)	
Yes	10,921 (21.9%)	18,717 (22.2%)	11,503 (20.5%)	7466 (22.0%)	9674 (24.6%)	
Not assessed	2870 (5.7%)	4283 (5.1%)	3207 (5.7%)	1745 (5.1%)	2243 (5.7%)	
Missing	787 (1.6%)	1259 (1.5%)	980 (1.7%)	627 (1.8%)	918 (2.3%)	
BMI (kg/m^2^), mean (SD)	20.6 (4.3)	20.9 (4.4)	20.7 (4.3)	20.9 (4.5)	21.1 (4.5)	<0.001
BMI categories						<0.001
Underweight	6877 (13.8%)	11,076 (13.1%)	6916 (12.3%)	3972 (11.7%)	4024 (10.2%)	
Normal	28,862 (57.8%)	48,279 (57.2%)	31,540 (56.1%)	18,932 (55.7%)	20,746 (52.8%)	
Overweight	5821 (11.7%)	10,874 (12.9%)	7068 (12.6%)	4468 (13.1%)	5413 (13.8%)	
Obese	2350 (4.7%)	4592 (5.4%)	2825 (5.0%)	1901 (5.6%)	2130 (5.4%)	
Missing	6007 (12.0%)	9570 (11.3%)	7895 (14.0%)	4739 (13.9%)	7013 (17.8%)	
Fast food consumption (times/day), mean (SD)	1.7 (1.4)	2.0 (1.5)	2.2 (1.6)	2.6 (1.7)	3.0 (2.1)	<0.001
Fruit consumption (times/day), mean (SD)	1.3 (1.3)	1.3 (1.2)	1.5 (1.3)	1.7 (1.4)	2.1 (1.8)	<0.001
Vegetable consumption (times/day), mean (SD)	1.6 (1.4)	1.6 (1.3)	1.7 (1.3)	1.8 (1.4)	2.1 (1.8)	<0.001

^1^ Data are presented as mean (SD) for continuous measures, and *n* (%) for categorical measures. *p* values are for group comparison.

**Table 2:** Odds ratio (95%CI) for aggressive behavior by soft drink consumption levels (*n* = 263,890) ^1^.

	None	<1 Time/Day	1 Time/Day	2 Times/Day	≥3 Times/Day	*p* Value for Trend
	*n* = 49,917	*n* = 84,391	*n* = 56,244	*n* = 34,012	*n* = 39,326	
Model 1	1.00	1.15 (1.12–1.19)	1.26 (1.22–1.30)	1.52 (1.46–1.57)	2.14 (2.06–2.21)	<0.001
Model 2	1.00	1.08 (1.05–1.12)	1.10 (1.06–1.14)	1.22 (1.17–1.27)	1.47 (1.42–1.53)	<0.001
Model 3	1.00	1.08 (1.05–1.12)	1.09 (1.06–1.13)	1.22 (1.17–1.26)	1.46 (1.41–1.52)	<0.001

^1^ Model 1 was adjusted for age and gender. Model 2 further adjusted for smoking, drinking, physical activity, intake of fruit, vegetable, and fast food. Model 3 further adjusted for BMI (underweight, normal, overweight, and obese).

**Table 3:** Association between soft drink consumption and aggressive behavior by gender, smoking, and physical and BMI status ^1^.

	None	<1 Time/Day	1 Time/Day	2 Times/Day	≥3 Times/Day	*p* for Interaction
*Gender*						
Men	1.00	1.09 (1.05–1.14)	1.13 (1.08–1.18)	1.24 (1.18–1.31)	1.53 (1.46–1.60)	0.207
Women	1.00	1.07 (1.02–1.13)	1.05 (0.99–1.11)	1.18 (1.11–1.26)	1.37 (1.29–1.45)	
*Smoking*						
Non-smoker	1.00	1.10 (1.06–1.14)	1.10 (1.06–1.15)	1.22 (1.16–1.28)	1.46 (1.40–1.53)	0.001
Smoker	1.00	0.93 (0.86–1.01)	1.02 (0.94–1.12)	1.03 (0.94–1.13)	1.28 (1.18–1.40)	
*Physical activity*						
Insufficient	1.00	1.06 (1.02–1.10)	1.04 (1.00–1.09)	1.18 (1.13–1.24)	1.45 (1.38–1.52)	<0.001
Sufficient	1.00	1.15 (1.08-1.23)	1.25 (1.16–1.34)	1.28 (1.19–1.39)	1.49 (1.38–1.61)	
*BMI status*						
Thin	1.00	1.11 (1.01–1.21)	1.06 (0.96–1.17)	1.19 (1.06–1.33)	1.58 (1.42–1.77)	0.475
Normal	1.00	1.11 (1.01–1.21)	1.06 (0.96–1.17)	1.19 (1.06–1.33)	1.58 (1.42–1.77)	
Overweight	1.00	1.06 (0.97–1.16)	1.10 (1.00–1.22)	1.21 (1.09–1.35)	1.49 (1.34–1.65)	
Obese	1.00	0.94 (0.82–1.08)	0.99 (0.85–1.15)	1.11 (0.94–1.30)	1.28 (1.10–1.50)	

^1^ Values were odds ratio (OR) (95% confidence interval (CI)). Models adjusted for age, gender, smoking, alcohol drinking, physical activity, food insecurity, and intake of fruit, vegetable, and fast food. Stratification variables were not adjusted in the corresponding models.

## Supplementary Materials

The following are available online at https://www.mdpi.com/2072-6643/12/3/694/s1, Figure S1: Prevalence of physical fight and mean soft drink consumption in each study. Figure S2: Sociodemographic and lifestyle determinants of frequent physical fight. Figure S3: Forest plot of the association between soft drink consumption and physical fight (Eastern Mediterranean Region; time/day). Figure S4: Forest plot of the association between soft drink consumption (time/day) and physical fight (South-East Asia Region). Figure S5: Forest plot of the association between soft drink consumption (time/day) and physical fight (Western Pacific Region). Figure S6: Forest plot of the association between soft drink consumption (time/day) and physical fight (African Region). Figure S7: Forest plot of the association between soft drink consumption (time/day) and physical fight (Region of Americas).
