# Supplementary material for: Association between Soft Drink Consumption and Aggressive Behaviour among a Quarter Million Adolescents from 64 Countries Based on the Global School-Based Student Health Survey (GSHS)
Source: Nutrients. 2020 Mar 5;12(3):694. doi: 10.3390/nu12030694 (PMC7146469; doi:10.3390/nu12030694)

Figure S1 Prevalence of aggressive behaviour and mean soft drink consumption in each study

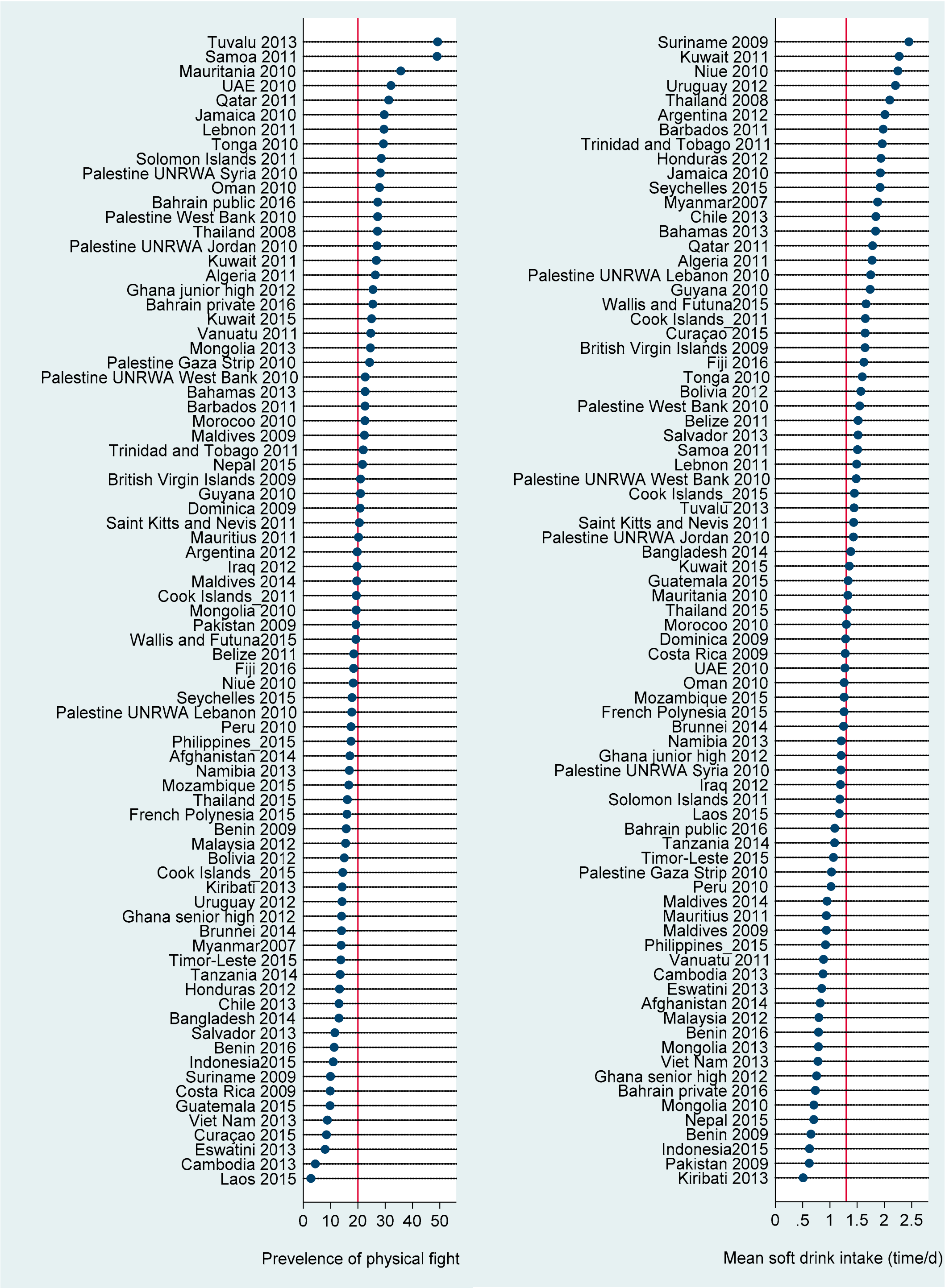

Values were calculated using svy command in Stata to take into account the survey weight.

Figure S2 Sociodemographic and lifestyle determinants of frequent aggressive behaviour

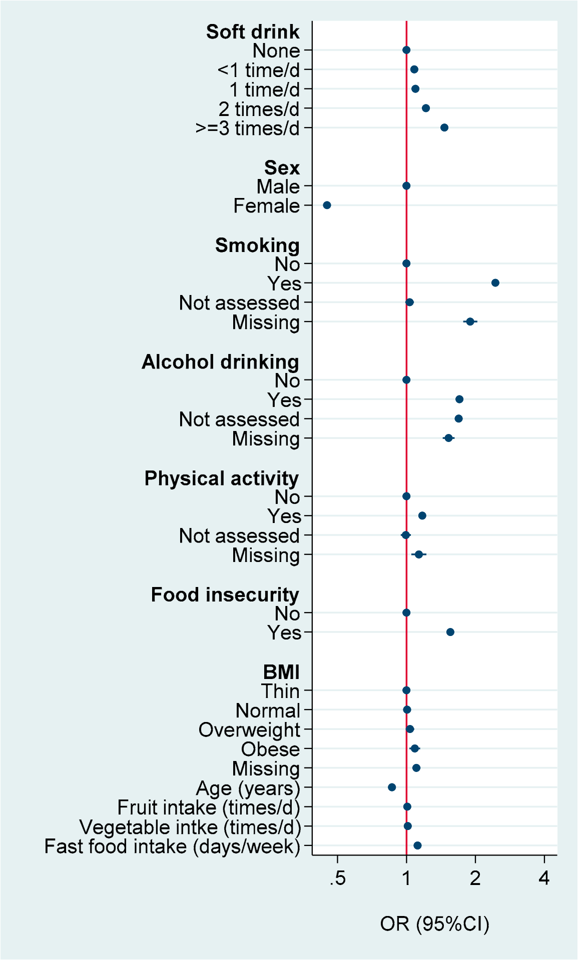

Values were odds ratio (95%CI) derived from logistic regression adjusting for all the variables in the figure.

Figure S3 Forest plot of the association between soft drink consumption and aggressive behaviour (Eastern Mediterranean Region) (time/day)

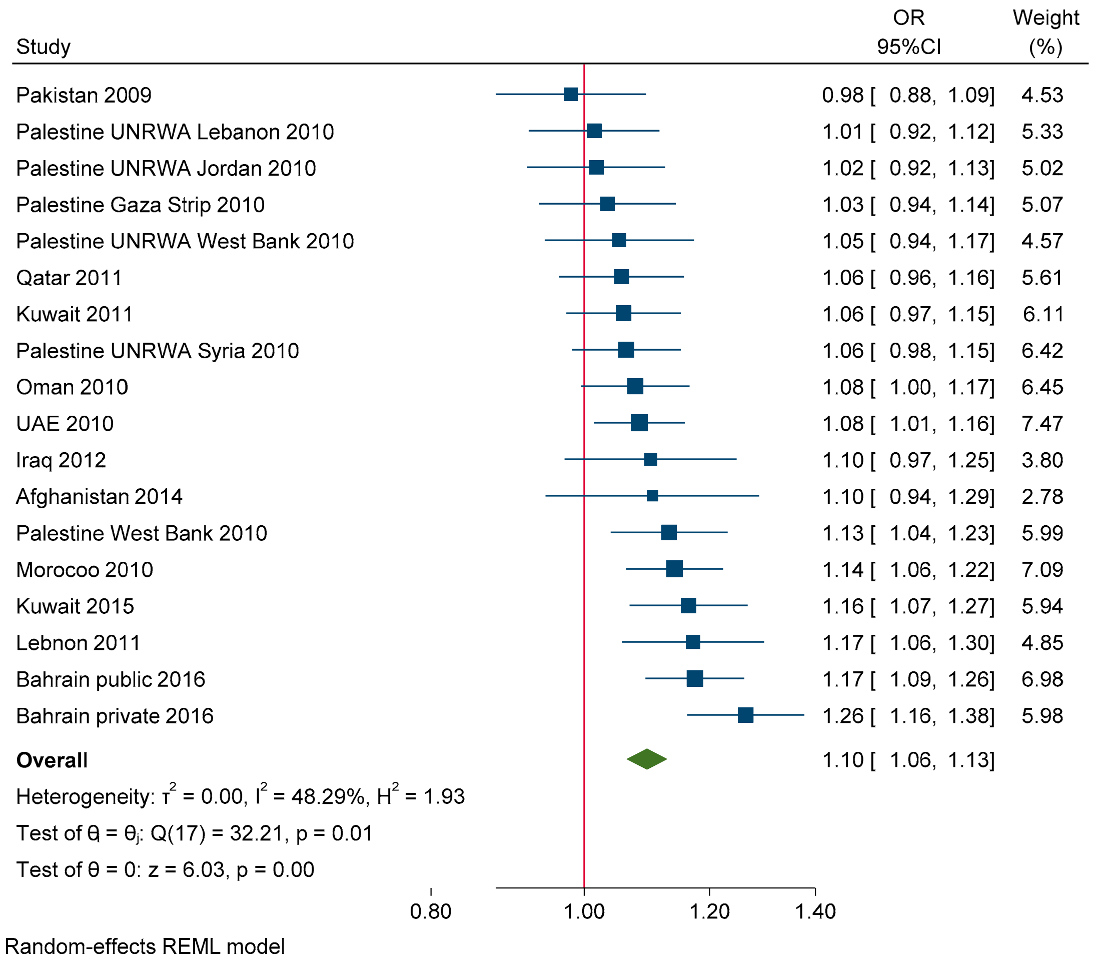

In each study, odds ratio was derived from logistic regression after adjusting for age, gender, smoking, alcohol drinking, sufficient physical activity, intake of fruit, vegetable and fast food, food insecurity. Survey weight was taken into consideration.

Figure S4 Forest plot of the association between soft drink consumption (time/day) and aggressive behaviour (South-East Asia Region)

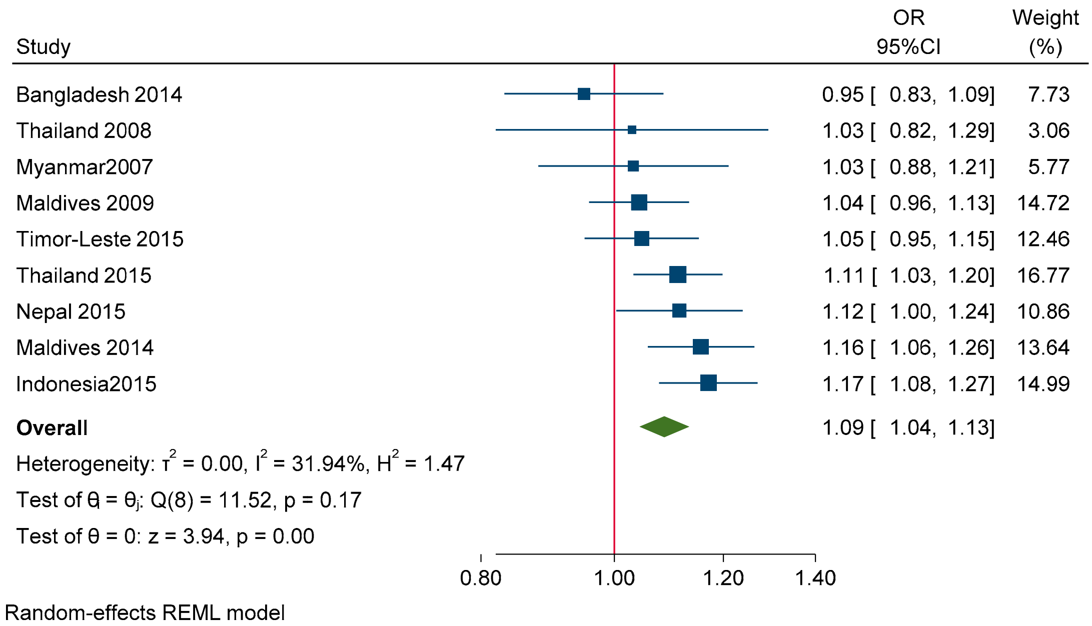

Figure S5 Forest plot of the association between soft drink consumption (time/day) and aggressive behaviour (Western Pacific Region)

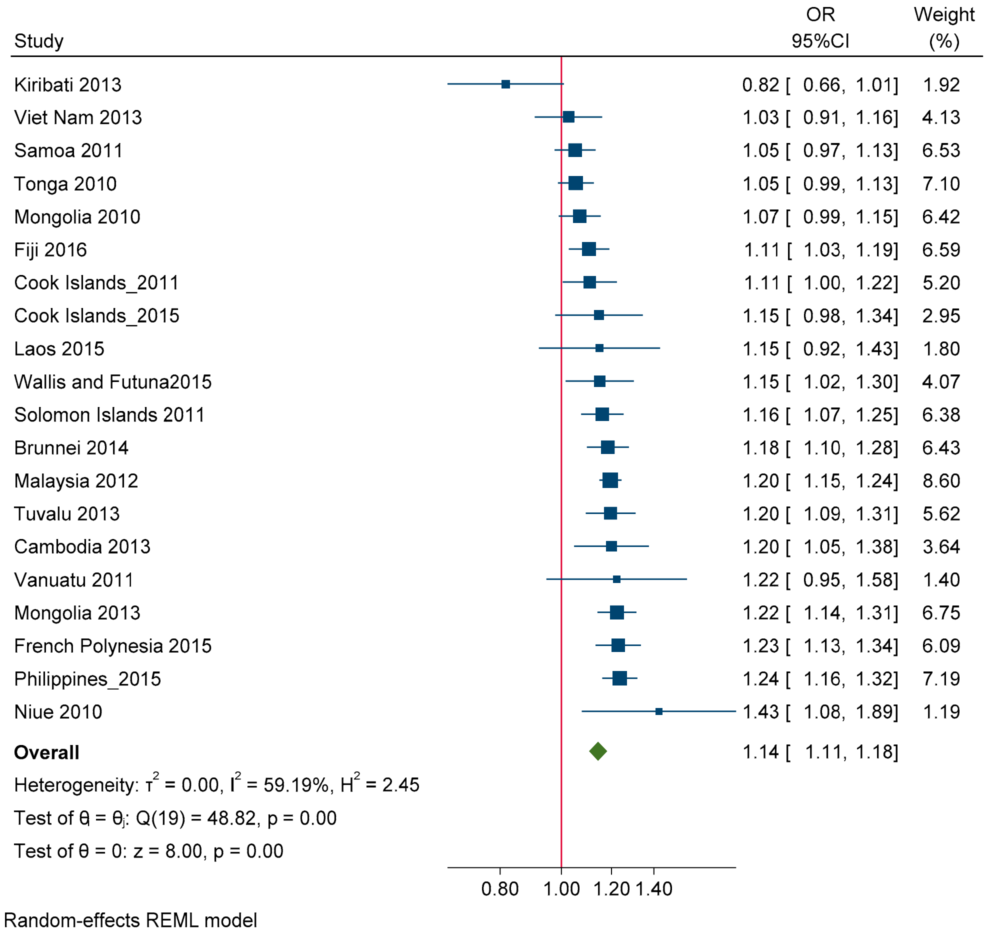

Figure S6 Forest plot of the association between soft drink consumption (time/day) and aggressive behaviour (African Region)

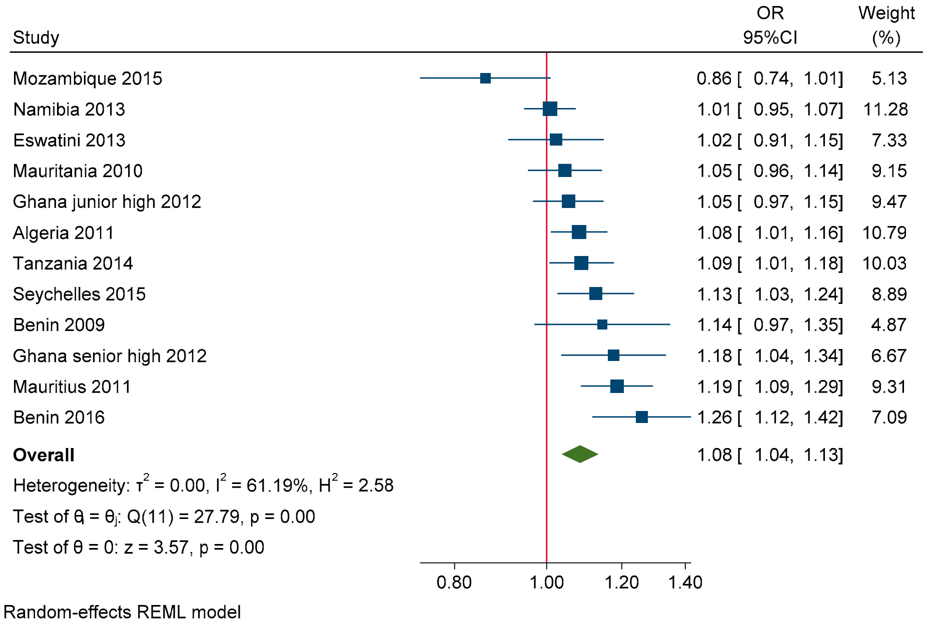

Figure S7 Forest plot of the association between soft drink consumption (time/day) and aggressive behaviour (Region of Americas)

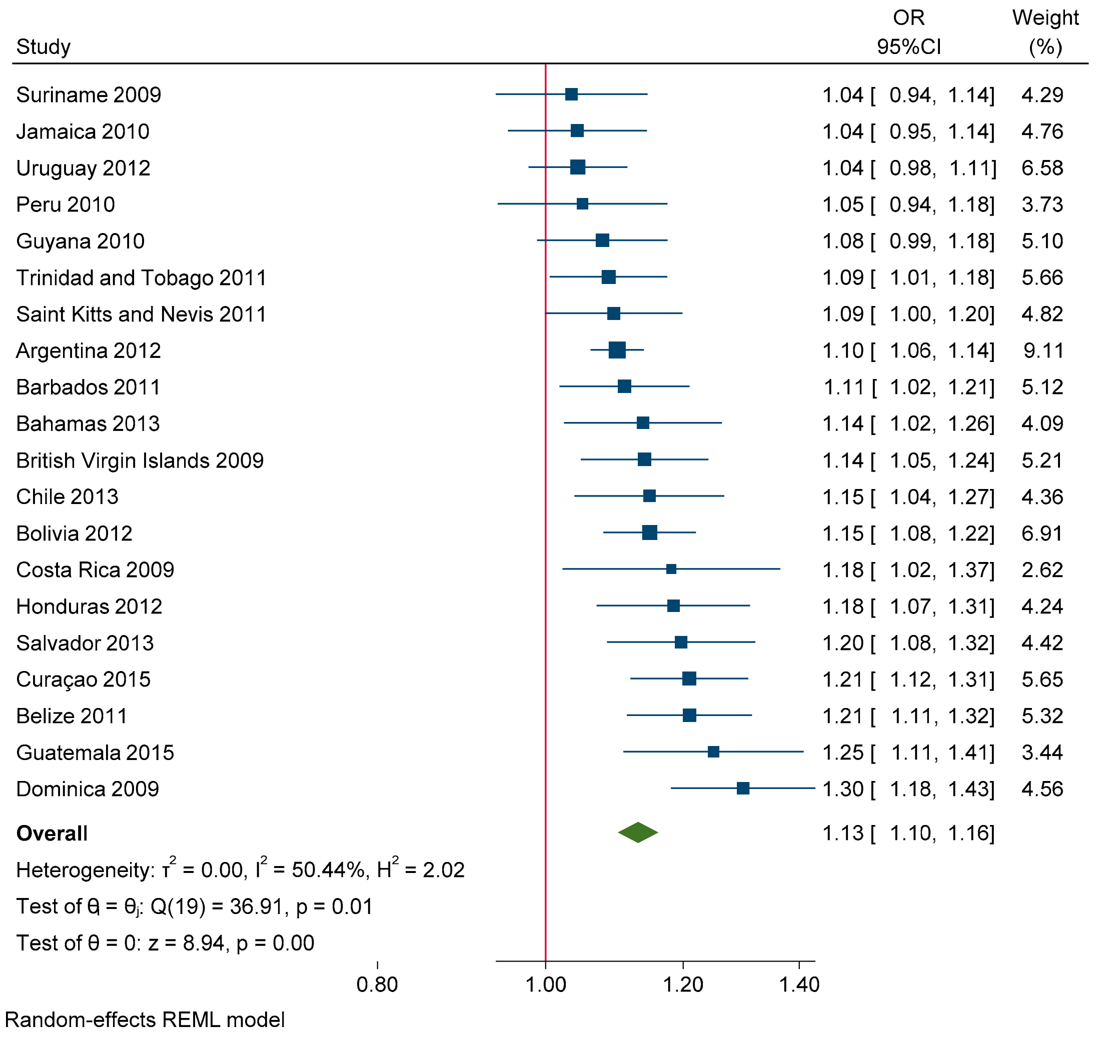

Supplement: Supplementary file 1 [file nutrients-12-00694-s001.pdf]
